# Supplementary material for: DNA Barcoding and Species Classification of Morchella
Source: Genes (Basel). 2022 Oct 6;13(10):1806. doi: 10.3390/genes13101806 (PMC9601616; doi:10.3390/genes13101806)
Supplement: Supplementary file 1 [file genes-13-01806-s001.zip › genes-1934528-supplementary.pdf]

Table S1. The information of single DNA molecualr barcoding sequences within *Morchella* genus

Species	Source	Sequences			
		EF1-α	***RPB1***	***RPB2***	***ITS***
Mel-13 M106	Xinjiang,China	MK965432	MK965479	MK965502	MK965444
Mel-13 M111	Xinjiang,China	MK965421	MK965468	MK965491	MK965445
Mel-13 M117	Xinjiang,China	MK965428	MK965475	MK965498	MK965446
Mel-13 M15	Xinjiang,China	MK965414	MK965461	MK965484	MK965437
Mel-13 M151	Xinjiang,China	MK965423	MK965470	MK965493	MK965449
Mel-13 M188	Xinjiang,China	MK965425	MK965472	MK965495	MK965450
Mel-13 M193	Xinjiang,China	MK965426	MK965473	MK965496	MK965451
Mel-13 M58	Xinjiang,China	MK965429	MK965476	MK965499	MK965447
Mel-19 M59	Xinjiang, China	MK965431	MK965478	MK965501	MK965454
Mel-19 M176	Xinjiang, China	MK965424	MK965471	MK965494	MK965457
Mel-19 M30	Xinjiang, China	MK965416	MK965463	MK965486	MK965439
Mel-19 M38	Xinjiang, China	MK965417	MK965464	MK965487	MK965440
Mel-19 M72	Xinjiang, China	MK965419_	MK965466	MK965489	MK965442
Mel-19 M74	Xinjiang, China	MK965420	MK965467	MK965490	MK965443
Mel-31 M192	Xinjiang, China	MK965430	MK965477	MK965500	MK965452
Mel-33 M144	Xinjiang, China	MK965422_	MK965469	MK965492	MK965448
Mel-33 M197	Xinjiang, China	MK965427	MK965474	MK965497	MK965456
Mel-33 M25	Xinjiang, China	MK965415	MK965462	MK965485	MK965438
Mel-33 M68	Xinjiang, China	MK965418	MK965465	MK965488	MK965441
M.eximioides OW-Mo2-17	Norway Buskerud, Ringerike, Vagard	MK639459	MK639551	MK639511	MK629409
M. norvegiensis ABO-Mo1-17	Norway	MK639468	MK639560	MK639520	MK629419

M. norvegiensis CRM-Mo1-17	Norway	MK639472	MK639561	MK639524	MK629423
M. norvegiensis LG-Mo1-17	Norway	MK639474	MK639562	MK639526	MK629425
M. norvegiensis IC-Mo1-17	Norway	MK639465	MK639557	MK639517	MK629416
M. norvegiensis IR-M1-15	Norway	MK639435	MK639532	MK639490	MK629378
M. norvegiensis JAS-Mo1-17	Norway	MK639460	MK639552	MK639512_	MK629410
M. norvegiensis JAS-Mo2-17	Norway	MK639461	MK639553	MK639513	MK629411
M. norvegiensis JAS-Mo3-17	Norway	MK639462	MK629412	MK639554	MK639512
M. norvegiensis MP-Mo-1-15	Norway	MK639431	MK639530	MK639488	MK629374
M. norvegiensis MP-Mo1-17	Norway	MK639467	MK639559	MK639519	MK629418
M. norvegiensis OK-Mo1-17	Norway	MK639466	MK639558	MK639518	MK629417
M. norvegiensis TJ-Mo1-15	Norway	MK639432	MK639531	MK639489	MK629375
M. eximioides LG-Mo1-17	Norway Sor-Trondelag, Trondheim, Charlottenlund	MK639463	MK639555	MK639515	MK629413
M. importuna EM-Mo1-17	Norway: Ostfold, Fredrikstad	MK639464	MK639556	MK639516	MK629415
M. clivicola 1022	Luoyang, Henan, China	MK321924	MK321892	MK321908	MK321876
M. clivicola 1021	Luoyang, Henan, China	MK321919	MK321887	MK321903	MK321871
M. clivicola 1020	Enshi, Hubei, China	MK321923	MK321891	MK321907	MK321875
M. confusa 1028	Mianyang, Sichuan, China	MK321867	MK321855	MK321861	MK321849
M. confusa 1027	Mianyang, Sichuan, China	MK321866	MK321854	MK321860	MK321848
M. owneri 1026	Baoding, Hebei, China	MK321865	MK321853	MK321859	MK321847
M. owneri 1025	Baoding, Hebei, China	MK321864	MK321852	MK321858	MK321846
M. palazonii 1032	Yili, Xinjiang, China	MK321932	MK321900	MK321916	MK321884
M. palazonii 1031	Yili, Xinjiang, China	MK321931	MK321899	MK321915	MK321883
M. yangii 1013	Luoyang, Henan, China	MK321933	MK321901	MK321917	MK321885
M. yangii 1012	Luoyang, Henan, China	MK321926	MK321894	MK321910	MK321878

M. yangii 1011	Luoyang, Henan, China	MK321922	MK321890	MK321906	MK321874
M. yangii 1014	Nanyang, Henan, China	MK321925	MK321893	MK321909	MK321877
M. yishuica 1017	Linyi, Shandong, China	MK321930	MK321898	MK321914	MK321882
M. yishuica 1016	Linyi, Shandong, China	MK321928	MK321896	MK321912	MK321880
M. yishuica 1015	Linyi, Shandong, China	MK321929	MK321897	MK321913	MK321881
*M. yishuic*a 1018	Linyi, Shandong, China	MK321927	MK321895	MK321911	MK321879
Mes-15 YAASM32	Sichuan, China	MG589686	MG598537	MG598579	MG589648
Mes-15 YAASM8	Yunnan, China	MG589682	MG598533	MG598575	MG589644
Mes-15 YAASMNHSQ	Yunnan, China	MG589706	MG598557	MG598599	MG589668
Mes-15 YAASM34	Yunnan, China	MG589687	MG598538	MG598580	MG589649
Mes-16 YAASM76	Yunnan, China	MG589698	MG598549	MG598591	MG589660
Mes-16 YAASMDL1	Yunnan, China	MG589704	MG598555	MG598597	MG589666
Mes-16 YAASMDL2	Yunnan, China	MG589705	MG598556	MG598598	MG589667
Mes-19 YAASM42	Sichuan, China	MG589689	MG598540	MG598582	MG589651
Mes-20 YAASM56	Hebei, China	MG589695	MG598546	MG598588	MG589657
Mes-20 YAASM57	Sichuan, China	MG589696	MG598547	MG598589	MG589658
Mes-20 YAASM58	Sichuan, China	MG589697	MG598548	MG598590	MG589659
Mes-20 YAASMCB1	Sichuan, China	MG589703	MG598554	MG598596	MG589665
Mes-25 YAASM14	Yunnan, China	MG589683	MG598534	MG598576	MG589645
Mes-25 YAASM15	Yunnan, China	MG589684	MG598535	MG598577	MG589646
Mes-6 YAASM49	Shanxi, China	MG589692	MG598543	MG598585	MG589654
Mes-6 YAASM80	Yunnan, China	MG589700	MG598551	MG598593	MG589662
Mes-9 YAASM46	Shandong, China	MG589690	MG598541	MG598583	MG589652
Mes-9 YAASM47	Shandong, China	MG589691	MG598542	MG598584	MG589653
M. importuna Cly-158	Sichuan, China	MG121863	MG121864	MG121865	MG121861

M. gracilis M686	Parque Nacional EI Avila	MH014704	MH014715	MH014718	MH014707
M. gracilis 9483 TJB	Dominican Republic	MH014703	MH014714	MH014717	MH014706
M. kaibabensis TAC 1708	USA Arizona	MH014722	MH014733	MH014738	MH014728
M. kaibabensis TAC 1376	USA Arizona	MH014721	MH014732	MH014737	MH014727
M. palazonii PhC149	Spain	MH781723	MH781725	MH781726	KT883899
M.exuberans KOD1548	Michigan	MF981032	MF981028	MF981030	MF981025
M.exuberans KOD1826	Tennessee	MF981031	MF981027	MF981029	MF981026
Mel-10 HKAS62868	Yunnan, China	JQ321842	JQ321938	JQ321970	JQ321874
Mel-10 HKAS62869	Yunnan, China	JQ321843	JQ321939	JQ321971	JQ321875
Mel-10 HKAS62870	Germany	JQ321870	JQ321966	JQ321998	JQ321902
Mel-10 HKAS62871	Germany	JQ321871	JQ321967	JQ321999	JQ321903
Mel-13 HKAS62887	Yunnan, China	JQ321841	JQ321937	JQ321969	JQ321873
Mel-13 HKAS62888	Sichuan, China	JQ321860	JQ321956	JQ321988	JQ321892
Mel-13 HKAS62889	Sichuan, China	JQ321852	JQ321948	JQ321980	JQ321884
Mel-13 HKAS62890	Shanxi, China	JQ321853	JQ321949	JQ321981	JQ321885
Mel-13 HKAS62891	Xinjiang, China	JQ321868	JQ321964	JQ321996	JQ321900
Mel-13 HKAS62892	Shanxi, China	JQ321854	JQ321950	JQ321982	JQ321886
Mel-13 HKAS62893	Yunnan, China	JQ321856	JQ321952	JQ321984	JQ321888
Mel-13 HKAS62894	Shanxi, China	JQ321872	JQ321968	JQ322000	JQ321904
Mel-14 HKAS62885	Sichuan, China	JQ321855	JQ321951	JQ321983	JQ321887
Mel-16 HKAS62883	Jilin, China	JQ321866	JQ321962	JQ321994	JQ321898
Mel-16 HKAS62884	Jilin, China	JQ321867	JQ321963	JQ321995	JQ321899
Mel-19 HKAS62873	Gansu, China	JQ321846	JQ321942	JQ321974	JQ321878
Mel-19 HKAS62875	Sichuan, China	JQ321858	JQ321954	JQ321986	JQ321890
Mel-20 HKAS62876	Yunnan, China	JQ321863	JQ321959	JQ321991	JQ321895

Mel-21 HKAS62878	Hubei, China	JQ321862	JQ321958	JQ321990	JQ321894
Mel-21 HKAS62879	Sichuan, China	JQ321851	JQ321947	JQ321979	JQ321883
Mel-21 HKAS62880	Sichuan, China	JQ321850	JQ321946	JQ321978	JQ321882
Mel-31 HKAS62881	Gansu, China	JQ321857	JQ321953	JQ321985	JQ321889
Mel-31 HKAS62882	Yunnan, China	JQ321865	JQ321961	JQ321993	JQ321897
Mel-33 HKAS62874	Gansu, China	JQ321861	JQ321957	JQ321989	JQ321893
Mel-6 HKAS62872	Yunnan, China	JQ321845	JQ321941	JQ321973	JQ321877
Mel-7 HKAS62863	Yunnan, China	JQ321869	JQ321965	JQ321997	JQ321901
Mel-7 HKAS62864	Yunnan, China	JQ321844	JQ321940	JQ321972	JQ321876
Mel-9 HKAS62865	Yunnan, China	JQ321847	JQ321943	JQ321975	JQ321879
Mel-9 HKAS62866	Yunnan, China	JQ321848	JQ321944	JQ321976	JQ321880
Mel-9 HKAS62867	Yunnan, China	JQ321849	JQ321945	JQ321977	JQ321881
Mes-15 HKAS55894	Sichuan, China	JQ322011	JQ322134	JQ322175	JQ322052
Mes-15 HKAS62913	Yunnan, China	JQ322005	JQ322128	JQ322169	JQ322046
Mes-15 HKAS62914	Yunnan, China	JQ322006	JQ322129	JQ322170	JQ322047
Mes-16 HAI-D-041	Israel	JQ322024	JQ322147	JQ322188	JQ322065
Mes-16 HKAS55839	Yunnan, China	JQ322007	JQ322130	JQ322171	JQ322048
Mes-16 HKAS55840	Yunnan, China	JQ322008	JQ322131	JQ322172	JQ322049
Mes-19 HKAS55910	Shaanxi, China	JQ322037	JQ322160	JQ322201	JQ322078
Mes-19 HKAS56568	Chongqing, China	JQ322021	JQ322144	JQ322185	JQ322062
Mes-20 HKAS55841	Yunnan, China	JQ322009	JQ322132	JQ322173	JQ322050
Mes-20 HKAS55842	Yunnan, China	JQ322010	JQ322133	JQ322174	JQ322051
Mes-21 HKAS55920	Shannxi, China	JQ322040	JQ322163	JQ322204	JQ322081
Mes-21 HKAS55921	Shannxi, China	JQ322038	JQ322161	JQ322202	JQ322079
Mes-22 HKAS55916	Zhejiang, China	JQ322016	JQ322139	JQ322180	JQ322057

Mes-22 HKAS55917	Zhejiang, China	JQ322017	JQ322140	JQ322181	JQ322058
Mes-22 HKAS55919	Zhejiang, China	JQ322018	JQ322141	JQ322182	JQ322059
Mes-23 HKAS56571	Chongqing, China	JQ322023	JQ322146	JQ322187	JQ322064
Mes-23HKAS62911	Anhui, China	JQ322001	JQ322124	JQ322165	JQ322042
Mes-25 HKAS62861	Sichuan, China	JQ322035	JQ322158	JQ322199	JQ322076
Mes-25 HKAS62862	Sichuan, China	JQ322036	JQ322159	JQ322200	JQ322077
Mes-26 HKAS55912	Hebei, China	JQ322014	JQ322137	JQ322178	JQ322055
Mes-26 HKAS55913	Hebei, China	JQ322015	JQ322138	JQ322179	JQ322056
Mes-27 HKAS55896	Sichuan, China	JQ322012	JQ322135	JQ322176	JQ322053
Mes-27 HKAS55897	Sichuan, China	JQ322013	JQ322136	JQ322177	JQ322054
Mes-6 HKAS56601	Shanxi, China	JQ322003	JQ322126	JQ322167	JQ322044
Mes-6 HKAS59162	Liaoning, China	JQ322031	JQ322154	JQ322195	JQ322072
Mes-6 HKAS59163	Liaoning, China	JQ322032	JQ322155	JQ322196	JQ322073
Mes-6 HMJAU5334	Jilin, China	JQ322041	JQ322164	JQ322205	JQ322082
Mes-6 HMJAU5454	Jilin, China	JQ322019	JQ322142	JQ322183	JQ322060
Mes-8 HKAS56676	Heilongjiang, China	JQ322020	JQ322143	JQ322184	JQ322061
Mes-8 HKAS59167	Liaoning, China	JQ322033	JQ322156	JQ322197	JQ322074
Mes-8 HKAS59168	Liaoning, China	JQ322034	JQ322157	JQ322198	JQ322075
Mes-9 HKAS59118	Shandong, China	JQ322025	JQ322148	JQ322189	JQ322066
Mes-9 HKAS59121	Shandong, China	JQ322026	JQ322149	JQ322190	JQ322067
Mes-9 HKAS59123	Shandong, China	JQ322027	JQ322150	JQ322191	JQ322068
Mes-9 HKAS59124	Shandong, China	JQ322028	JQ322151	JQ322192	JQ322069
Mes-9 HKAS59128	Shandong, China	JQ322029	JQ322152	JQ322193	JQ322070
Mel-12 HT299	CA-USA California	JN085092	JN085208	JN085264	JN085149
Mel-12 HT699	OR-USA Oregon	JN085104	JN085220	JN085276	JN085161

Mel-13 HT184	India	JN085090	JN085206	JN085262	JN085146
Mel-13 HT424	India	JN085101	JN085217	JN085273	JN085158
Mel-13 HT426	Kars (Q) Eastern Anatolia	JN085059	JN085175	JN085231	JN085115
Mel-13 HT815	China	JN084947	JN084999	JN085052	JN085167
Mel-14 HT725	China	JN085106	JN085222	JN085278	JN085163
Mel-14 HT728	China	JN085107	JN085223	JN085279	JN085164
Mel-15 HT307	VA-USA Virginia	JN085095	JN085211	JN085267	JN085152
Mel-15 HT407	OH-USA Ohio	JN085094	JN085210	JN085266	JN085151
Mel-16 HT489	Norway	JN085097	JN085213	JN085269	JN085154
Mel-16 M539	Denmark	JN085102	JN085218	JN085274	JN085159
Mel-16 S F13634	Sweden, Uppland	JN085053	JN085169	JN085225	JN085109
Mel-16 S F60062	Sweden, Södermanland	JN085082	JN085198	JN085254	JN085138
Mel-19 M218	Sweden	JN085088	JN085204	JN085260	JN085144
Mel-19 S F46072	Sweden	JN085065	JN085181	JN085237	JN085121
Mel-19HT735	China	JN085108	JN085224	JN085280	JN085165
Mel-19S_F99479	Sweden	JN085083	JN085199	JN085255	JN085139
Mel-20 HT195	Mugla Agean	JN085086	JN085202	JN085258	JN085142
Mel-20 HT214	Sweden	JN085087	JN085203	JN085259	JN085143
Mel-20 HT297	Konya (G) Central Anatolia	JN085055	JN085171	JN085227	JN085111
Mel-20 HT453	Konya (G) Central Anatolia	JN085063	JN085179	JN085235	JN085119
Mel-20 HT814	China	JN084946	JN084998	JN085051	JN085166
Mel-20HT456	Konya (G) Central Anatolia	JN085064	JN085180	JN085236	JN085120
Mel-20HT474	Taiwan	JN085098	JN085214	JN085270	JN085155
Mel-21 HT222	Japan	JN085091	JN085207	JN085263	JN085148
Mel-21 M225	Japan	JN085099	JN085215	JN085271	JN085156

Mel-22 HT431	CA-USA California	JN085093	JN085209	JN085265	JN085150
Mel-22 HT839	OR-USA Oregon	JN085105	JN085221	JN085277	JN085162
Mel-23 M495	Norway	JN085096	JN085212	JN085268	JN085153
Mel-23HT542	Denmark	JN085103	JN085219	JN085275	JN085160
Mel-25 HT436	Yozgat Central Anatolia	JN085061	JN085177	JN085233	JN085117
Mel-25 HT488	Çanakkale (A) Marmara	JN085072	JN085188	JN085244	JN085128
Mel-25 HT495	Çanakkale (A) Marmara	JN085073	JN085189	JN085245	JN085129
Mel-25 HT539	Mersin (H) Mediterranean	JN085080	JN085196	JN085252	JN085136
Mel-25 HT540	Mersin (H) Mediterranean	JN085081	JN085197	JN085253	JN085137
Mel-26 HT508	Yozgat (L) Central Anatolia	JN085075	JN085191	JN085247	JN085131
Mel-26 HT509	Yozgat (L) Central Anatolia	JN085076	JN085192	JN085248	JN085132
Mel-26 S F27848	Sweden	JN085054	JN085170	JN085226	JN085110
Mel-27 HT448	Konya (G) Central Anatolia	JN085062	JN085178	JN085234	JN085118
Mel-27 HT478	Kayseri (K) Central Anatolia	JN085070	JN085186	JN085242	JN085126
Mel-27 HT511	Kahramanmaras Mediterranean	JN085078	JN085194	JN085250	JN085134
Mel-27 HT520	Adana Mediterranean	JN085079	JN085195	JN085251	JN085135
Mel-28 HT401	Yozgat (L) Central Anatolia	JN085058	JN085174	JN085230	JN085114
Mel-28 HT507	Yozgat (L) Central Anatolia	JN085074	JN085190	JN085246	JN085130
Mel-28 HT510	Yozgat (L) Central Anatolia	JN085077	JN085193	JN085249	JN085133
Mel-29 HT470	Uşak (E) Aegean	JN085066	JN085182	JN085238	JN085122
Mel-29 HT471	Uşak (E) Aegean	JN085067	JN085183	JN085239	JN085123
Mel-30 HT193	Muğla (C) Agean	JN085085	JN085201	JN085257	JN085141
Mel-31 HT354	Black Sea Kastamonu	JN085056	JN085172	JN085228	JN085112
Mel-31 HT396	Kahramanmaraş (J) Mediterranean	JN085057	JN085173	JN085229	JN085113
Mel-31 HT472	Us¸ak Mediterranean	JN085068	JN085184	JN085240	JN085124

Mel-32 HT106	Kastamonu Black Sea	JN085084	JN085200	JN085256	JN085140
Mel-32 HT428	Kars East Anatolian	JN085060	JN085176	JN085232	JN085116
Mel-32 HT477	Kayseri Central Anatolia	JN085069	JN085185	JN085241	JN085125
Mel-32 HT479	Kayseri Central Anatolia	JN085071	JN085187	JN085243	JN085127
Mel-20 HT 123	Kastamonu Black Sea	HM056315	HM056413	HM056475	HM056376
Mel-20 HT 165	Denizli Agean	HM056323	HM056421	HM056483	HM056381
Mel-20 HT 168	Denizli Agean	HM056325	HM056423	HM056485	HM056382
Mel-20 HT 181	Mediterranean	HM056328	HM056426	HM056488	HM056383
Mel-20 HT 182	Adana Mediterranean	HM056329	HM056427	HM056489	HM056384
Mel-20 HT 183	Mediterranean	HM056330	HM056428	HM056490	HM056385
Mel-20 HT 184	Mediterranean	HM056331	HM056429	HM056491	HM056386
Mel-20 HT 187	Mugˇla Agean	HM056332	HM056430	HM056492	HM056387
Mel-20 HT 191	Mugla Agean	HM056334	HM056432	HM056494	HM056389
Mel-20 HT 195	Mugla Agean	HM056336	HM056434	HM056496	HM056391
Mel-20 HT 260	Sivas (I) Central Anatolian	HM056359	HM056457	HM056519	HM056399
Mel-20 HT 42	Mediterranean	HM056360	HM056458	HM056520	HM056400
Mel-20 HT 46	Mediterranean	HM056362	HM056460	HM056522	HM056402
Mel-20 HT 92	Unknown	HM056367	HM056465	HM056527	HM056406
Mel-25 HT 157	Aydın Agean	HM056322	HM056420	HM056482	HM056380
Mel-25 HT 188	Mugˇla Agean	HM056333	HM056431	HM056493	HM056388
Mel-25 HT 65	Mugˇla Agean	HM056364	HM056462	HM056524	HM056404
Mel-26 HT 120	Kastamonu Black Sea	HM056313	HM056411	HM056473	HM056374
Mel-26 HT 122	Kastamonu Black Sea	HM056314	HM056412	HM056474	HM056375
Mel-26 HT 124	Kastamonu Black Sea	HM056316	HM056414	HM056476	HM056377
Mel-26 HT 156	Aydın Agean	HM056321	HM056419	HM056481	HM056379

Mel-27 HT 107	Kastamonu Black Sea	HM056310	HM056408	HM056470	HM056372
Mel-27 HT 118	Kastamonu Black Sea	HM056312	HM056410	HM056472	HM056373
Mel-27 HT 207	Kahramanmaras(G) Mediterranean	HM056343	HM056441	HM056503	HM056395
Mel-27 HT 25	Mersin (E) Mediterranean	HM056357	HM056455	HM056517	HM056397
Mel-29 HT 155	Aydın Agean	HM056320	HM056418	HM056480	HM056378
Mel-29 HT 45	Adana Mediterranean	HM056361	HM056459	HM056521	HM056401
Mel-29 HT 82	Mersin (E) Mediterranean	HM056365	HM056463	HM056525	HM056405
Mel-30 HT 193	Muğla (C) Agean	HM056335	HM056433	HM056495	HM056390
Mel-31 HT 202	Kahramanmaras Mediterranean	HM056341	HM056439	HM056501	HM056393
Mel-31 HT 203	Kahramanmaras Mediterranean	HM056342	HM056440	HM056502	HM056394
Mel-31 HT 212	Kahramanmaras Mediterranean	HM056344	HM056442	HM056504	HM056396
Mel-8 HT 251	Unknown	HM056358	HM056456	HM056518	HM056398
Mel-8 HT 50	Unknown	HM056363	HM056461	HM056523	HM056403
M. brunnea Mel Dung 02-04	Whitman County Washington	KM204663	KM204727	KM204754	KM204686
M. brunnea 11-29-Dung	Whitman County Washington	KM204662	KM204728	KM204755	KM204687
M. snyderi MM 4	Latah County Idaho	KM034930	KM204724	KM204750	KM204682
M. snyderi MM 2-1A	Latah County Idaho	KM034929	KM204723	KM204747	KM204681
M. snyderi 11-12-VM 1-4	Latah County Idaho	KM204763	KM204726	KM204748	KM204678
M. snyderi Umatilla Black 01-01	Garfield County Washington	KM034927	KM204722	KM204751	KM204679
M. snyderi Dung 04-03	Garfield County Washington	KM034926	KM204721	KM204752	KM204683
M. snyderi ARD 04-02	Latah County Idaho	KM034925	KM204720	KM204753	KM204677
M. snyderi 11-12-VM 1-2	Latah County Idaho	KM204764	KM204766	KM204767	KM204768
M. snyderi 11-12-VM 1-5	Latah County Idaho	KM034928	KM204725	KM204749	KM204680
Mel-37 CIEFAP5	Chubut:PNLA Senda arroyo Cascada	KJ569626	KJ569594	KJ569620	KJ439678
Mel-37 CIEFAP57	Chubut:Trevelin, La 106 Farm	KJ569627	KJ569593	KJ569621	KJ439670

Mel-37 CIEFAP69	Chubut:Lago Puelo, Río Azul catwalk	KJ569629	KJ569595	KJ569623	KJ439677
Mel-37 CIEFAP71	Chubut:Lago Puelo, Río Azul catwalk	KJ569630	KJ569596	KJ569624	KJ439673
Mel-37 CIEFAP74	Chubut:Lago Puelo, Río Azul catwalk	KJ569631	KJ569598	KJ569625	KJ439674
MKH3B	Marco River Forest Farm，Qinghai,China				
m111	Marco River Forest Farm，Qinghai,China				
M118	Marco River Forest Farm，Qinghai,China				
M148	Marco River Forest Farm，Qinghai,China				
M200	Marco River Forest Farm，Qinghai,China				
M34	Marco River Forest Farm，Qinghai,China				
M35	Marco River Forest Farm，Qinghai,China				
M69	Marco River Forest Farm，Qinghai,China				
M73	Marco River Forest Farm，Qinghai,China				
10号	Xining,Qinghai,China				
14号	Xining,Qinghai,China				
16号	Xining,Qinghai.China				

The information of *Morchella* species, including species names, source and accession numbers

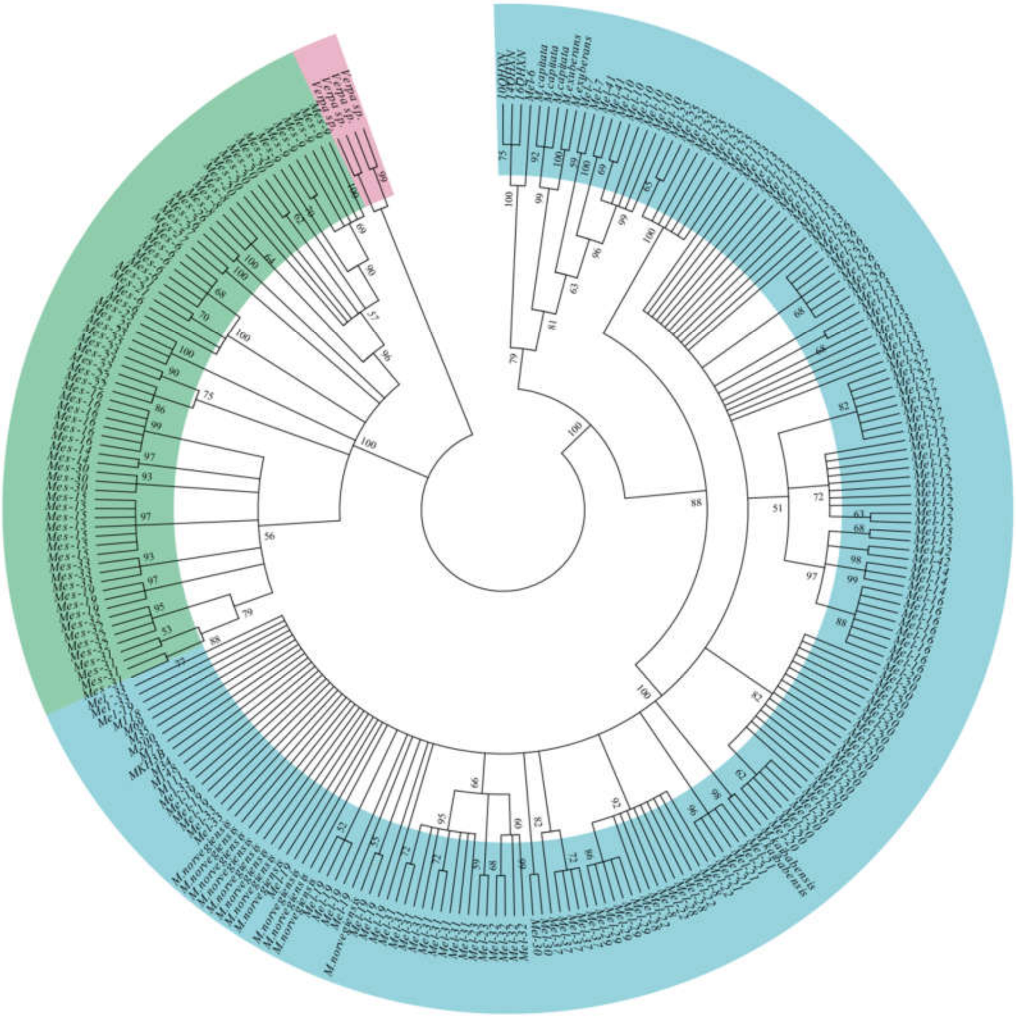

Figure S1 Maximum Parsimony phylogenetic tree of *Morchella* based on the combined (ITS, *EF1-α*, *RPB1* and *RPB2*) dataset totaled 1578bp of aligned DNA sequences, the *Verpa* sp. was used as outgroup. And the bootstrap support values based on 1000 pseudoreplicates are indicated above the branches, and the support rates > 50% . Pink stands for the outgroup, green represents Esculenta clade, and blue is Elata clade.

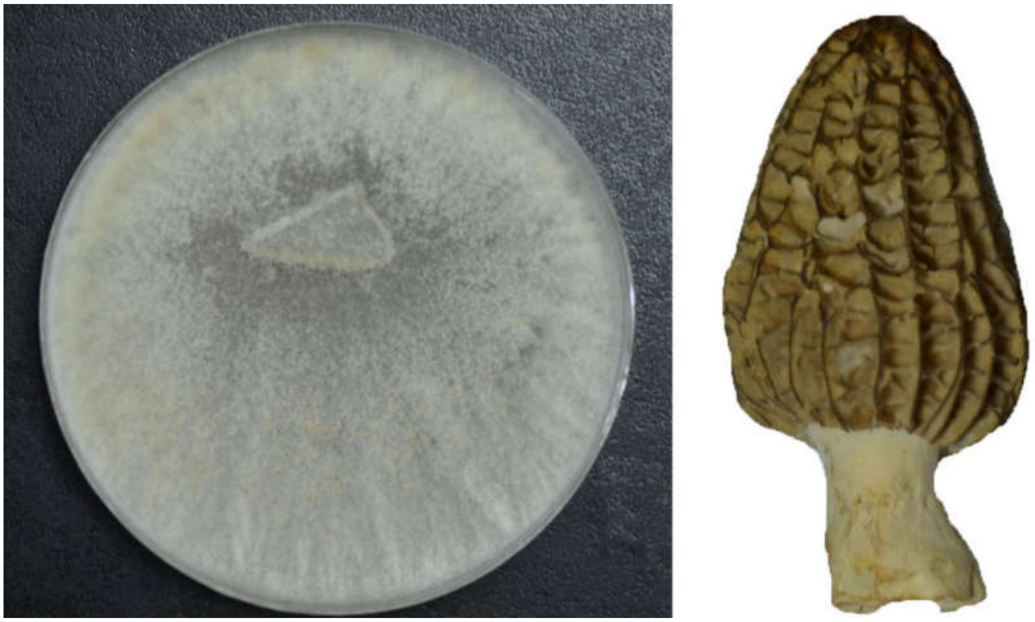

Figure S2. The picture of *Morchella* mycelium and strain.

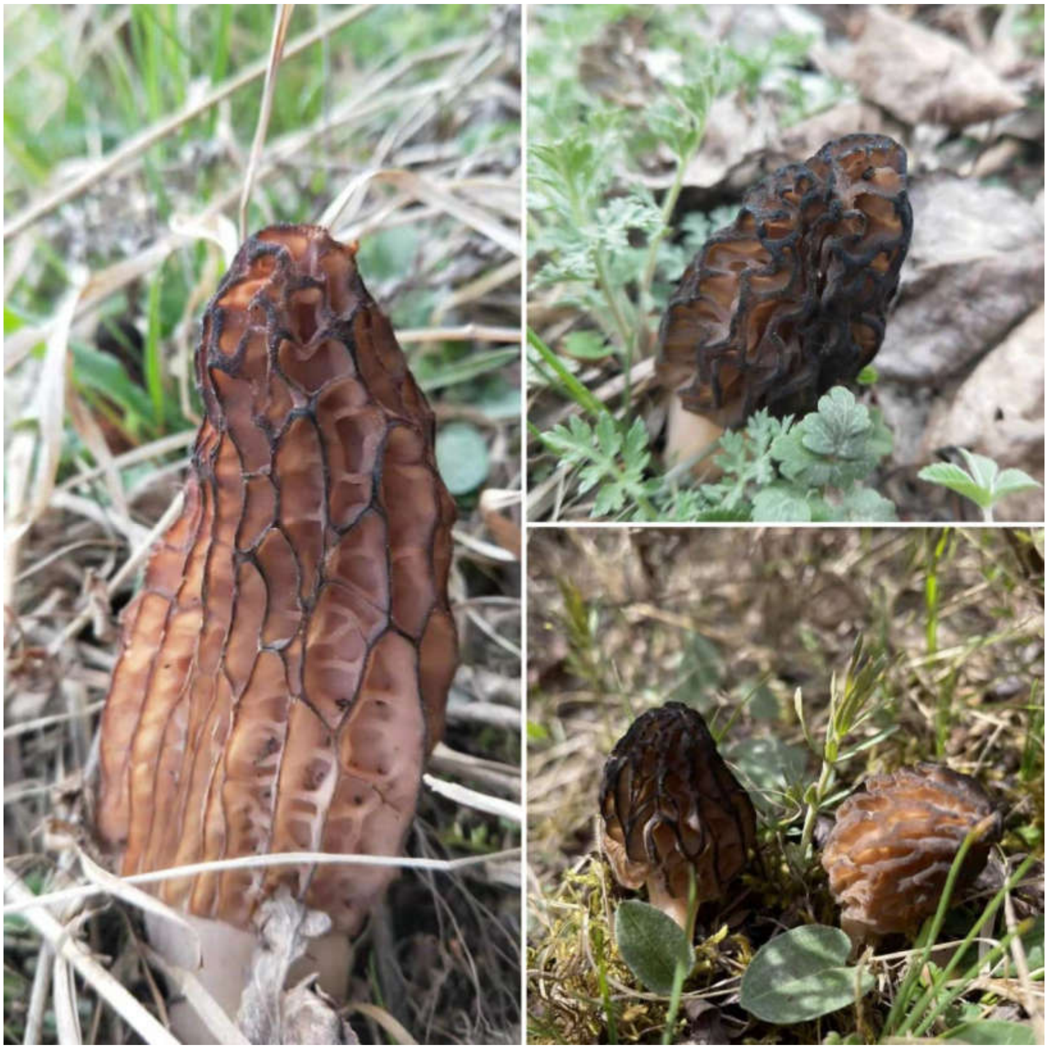

Figure S3 The image of wild morels habitat.
